# High-Volume Hospitals’ Ovarian Cancer Care—Less Individual Approach or Better Treatment Results?

**DOI:** 10.3390/curroncol29080419

**Published:** 2022-07-26

**Authors:** Sonja Millert-Kalińska, Dominik Pruski, Marcin Przybylski, Małgorzata Stawicka-Niełacna, Edyta Mądry, Radosław Mądry

**Affiliations:** 1 Doctoral School, Poznan University of Medical Sciences, 61-701 Poznań, Poland; 2 Department of Obstetrics and Gynecology, District Public Hospital in Poznan, 60-479 Poznań, Poland; dominik.pruski@icloud.com (D.P.); nicramp@poczta.onet.pl (M.P.); 3 Department of Clinical Genetics and Pathology, University of Zielona Góra, 65-046 Zielona Góra, Poland; m.stawicka-nielacna@cm.uz.zgora.pl; 4 Department of Physiology, Poznan University of Medical Sciences, 61-701 Poznań, Poland; emadry@ump.edu.pl; 5 Department of Oncology, Poznan University of Medical Sciences, 60-569 Poznań, Poland; radoslaw.madry@skpp.edu.pl

**Keywords:** epithelial ovarian cancer, high-volume hospital, oncological units, laparoscopy in oncology

## Abstract

Ovarian cancer (OC) is the eighth most common cancer worldwide and is usually diagnosed in advanced stages. The relationship between treatment in high-volume hospitals (HVHs) and survival in OC has been documented by multiple studies, which showed that superior treatment and survival outcomes are associated with surgical expertise and multidisciplinary resources. To our study, 135 first-time patients treated in the years 2019–2020 in the Department of Oncology of Poznań University of Medical Sciences were enrolled. Th analysis showed a significant dependency between being treated in a HVH from the beginning of one’s diagnosis and the scope of the first intervention. Additionally, among patients treated in our centre, a significant portion of patients underwent laparoscopy, and from one year to another the number of laparoscopies performed increased. This may indicate that more patients began to qualify for neoadjuvant treatment. Patients benefit the most from surgery in a centre with more experience in treating ovarian cancer. In the future, we will be able to expand this study by using data from patients treated before 2019 and analysing larger cohorts of patients. This might enable us to update the rates of overall survival (OS), objective response rate (ORR) and progression-free survival (PFS).

## 1. Introduction

Ovarian cancer (OC) is the eighth most common cancer worldwide. According to GLOBOCAN, estimates suggest that in 2020, over 300,000 new cases of ovarian cancer were documented, whereas over 200,000 deaths were confirmed. In terms of morbidity and mortality rates, ovarian cancer ranks eighth in the world—3.4% and 4.7%, respectively. As reported by the National Cancer Registry published annually in Poland, in 2019, OC was the second most common gynaecological cancer (4.3%) after endometrial cancer, whereas it was the most common cause of death (6.0%). For over a decade, the trend observed in Poland has remained constant: in 2019, 3710 new cases of ovarian cancer were recorded. In line with the literature, about 70% of ovarian cancer cases are diagnosed in the advanced stages, associated with a low 5-year survival rate. According to the International Federation of Gynaecology and Obstetrics (FIGO), the 5-year survival rate in patients with OC diagnosed in stage III and IV is 30–50%, whereas for those with OC diagnosed in stage I and II, it is 80–90% [1]. A patient’s survival depends on, among other things, the histological type of the tumour, the stage of FIGO grading, the scope of cytoreductive surgery and the residual disease. Although the most common ovarian cancer susceptibility genes are BRCA1 and BRCA2, advancements in next-generation sequencing (NGS) analysis technology enabled the discovery of several non-BRCA genes responsible for OC. Current studies are focusing on the determination of the predisposition to developing cancer and the consideration of treatments for cancer, prevention strategies, risk diagnosis methods and the adoption of preventive measures for relatives.

Clinicians might manage therapy by considering the time of relapse and the toxicity profile associated with the side effects of previous lines of treatment. The currently proposed regimens require attending physicians to be prudent based on their experience. Therefore, the long-term monitoring of patients continuing treatment in one centre is essential.

The relationship between treatments in high-volume hospitals (HVHs) and survival in ovarian cancer has been documented by multiple population-based studies, which showed that superior treatment and survival outcomes are associated with surgical expertise and multidisciplinary resources offered by both high-volume physicians and hospitals. Therefore, we present a small but reliable analysis: the two-year observation of patients treated in one centre. In our work, we compared the treatment results of patients with OC in one centre with those treated in different centres. We looked for factors that may have affected this result, and we wanted to check whether treatment in oncologic units is more beneficial. The determination of specific variables may, in the future, influence the therapeutic decisions of clinicians and the choice where the multifaceted treatment of patients should be carried out.

## 2. Materials and Methods

Our study received a positive opinion from the Bioethics Committee of the Poznań University of Medical Sciences regarding the lack of a medical experiment and its retrospective nature. We present a retrospective observational study that was conducted in the Clinical Hospital of the University of Medical Sciences in Poznań, Poland (abbreviated to SKPP). The material consisted of patients treated in the Department of Gynaecological Oncology due to ovarian cancer. We carried out a detailed, multifaceted analysis of patients admitted to the ward for the first time in 2019–2020. First, we wanted to determine the FIGO profile of each patient and present the percentage of the prevalence of each histological type of ovarian cancer treated in our clinic. Then, we analysed the ratio of the “one roof patients” and the migration rate between different oncology centres. Subsequently, we assessed the scope of the first intervention and its radical nature—primary debulking surgery (PDS), neoadjuvant chemotherapy (NACT) and laparoscopy (LAP). Finally, we analysed the known mutations occurring in a particular group of patients using next-generation sequencing (NGS) technology.

**Figure 1:**
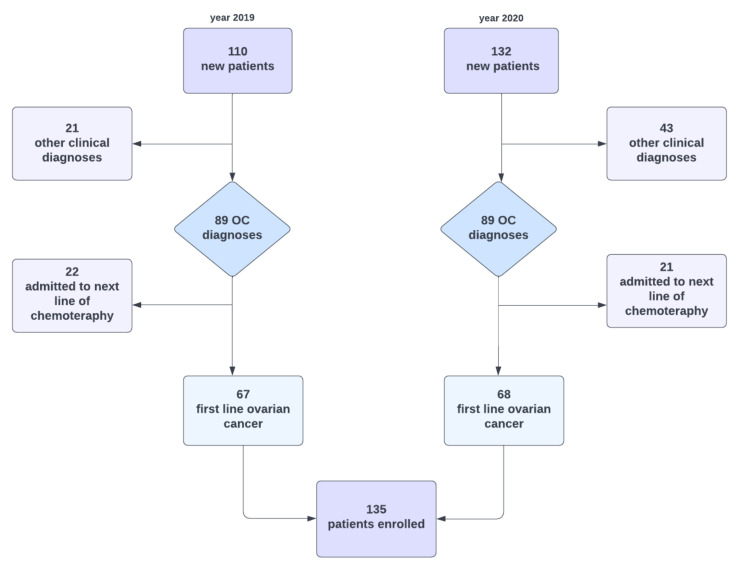
Flow-chart. OC—ovarian cancer. The inclusion criteria: (i) the histopathological confirmation of ovarian cancer based on surgery with laparotomy, laparoscopy or biopsy, (ii) over 18 years of age, (iii) not pregnant and (iv) written consent to the proposed treatment, including both surgical treatment and subsequent chemotherapy.

**Figure 2:**
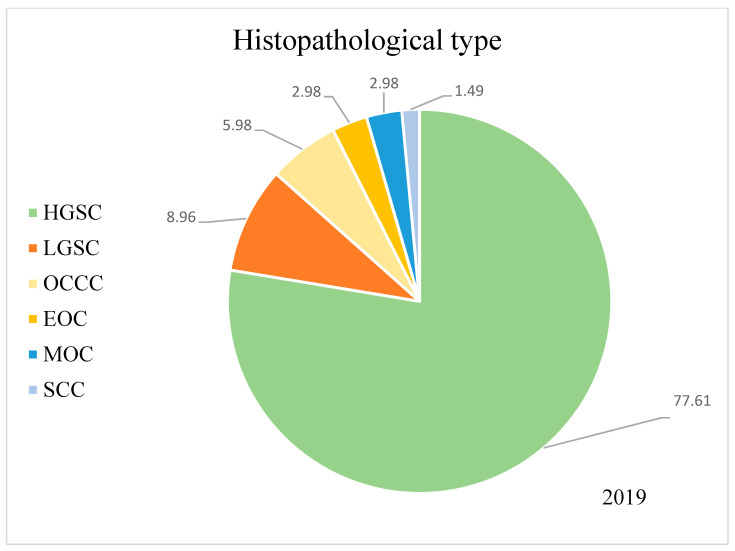
Histopathological types of OC in patients admitted in the year 2019. HGSC—high-grade serous carcinoma; LGSC—low-grade serous carcinoma; OCCC—ovarian clear-cell carcinoma; EOC—endometrioid ovarian cancer; MOC—mucinous ovarian cancer; SCC—squamous cell carcinoma.

**Figure 3:**
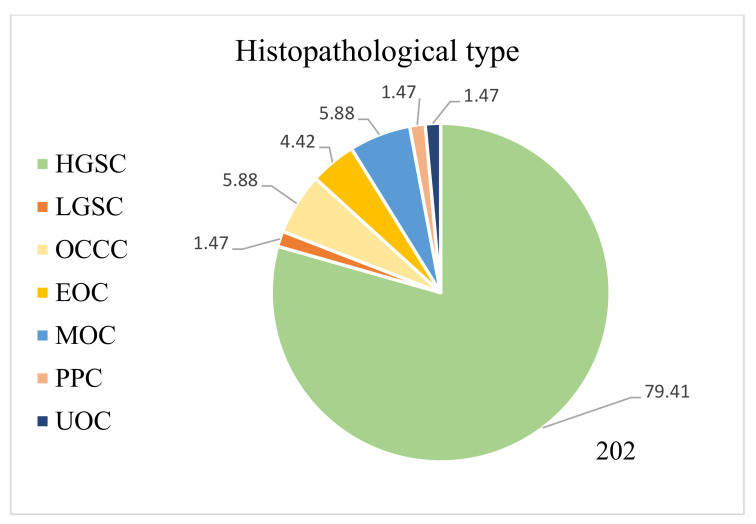
Histopathological types of OC in patients admitted in the year 2020. HGSC—high-grade serous carcinoma; LGSC—low-grade serous carcinoma; OCCC—ovarian clear-cell carcinoma; EOC—endometrioid ovarian cancer; MOC—mucinous ovarian cancer; PPC—primary peritoneal cancer; UOC—undifferentiated carcinoma of the ovary.

Our study initially analysed patients referred to the Department of Gynaecological Oncology ward in 2019–2020 with suspected ovarian cancer. The inclusion criteria were as follows: (i) the histopathological confirmation of ovarian cancer based on surgery with laparotomy, laparoscopy or biopsy, (ii) over 18 years of age, (iii) not pregnant and (iv) written consent to the proposed treatment, including both surgical treatment and subsequent chemotherapy. The exclusion criteria were: (i) another confirmed histopathological diagnosis, including intestinal tumours, tumours of the genital organs not derived from the ovary and benign lesions and (ii) patients transferred from other centres for the purpose of conducting subsequent lines of chemotherapy treatment. Patients who did not qualify for a specific type of chemotherapy due to their poor general condition and who did not accept the proposed treatment were rejected. The final exclusion criterion was patients undergoing fertility-conserving treatment (FCT). Figure 1 presents the process of recruiting patients for the study, and Figure 2 and Figure 3 show the percentage breakdowns of histopathological types of OC (*n* = 135).

## 3. Statistical Analysis

We conducted all analyses in the statistical software R, version 4.2.1 (R Core Team 2022, R: language and environment for statistical computing by the R Foundation for Statistical Computing, Vienna, Austria). Nominal variables are presented in the tables as numbers and percent of observation, whereas quantitative variables are presented as the medians of quartile 1 and 3 (normality of distribution was determined with the Shapiro–Wilk test). Dependencies between two variables were analysed with the chi-square test (with Yate’s correction for continuity for 2 × 2 tables) or Fisher’s exact test. A series of univariate cox regression analyses was conducted; hazard ratios with 95% confidence intervals and *p*-values are presented in the tables. A multivariate cox regression model was built using a step method, where variables with a *p*-value less than 0.250 in univariate models were predictors in the initial multivariate model [2]. The VIF value was checked while building the final multivariate model. Relapse-free survival curves were shown for all patients and broken down into smaller groups. Log-rank tests were conducted to determine the differences in the relapse rates between groups.

## 4. Results

In 2019, 110 subjects reported to the Department of Gynaecological Oncology and were examined. Due to the study’s criteria, we rejected 22 women admitted to follow the treatment line of ovarian cancer and 21 diagnosed with a different clinical diagnosis. In 2020, we registered 132 new hospital admissions; however, we rejected 21 and 43 patients, respectively. After screening, 135 subjects fulfilled all of the inclusion criteria and fulfilled none of the exclusion criteria. At diagnosis, the median age was 60 years (range = 36–88 years), and 104 patients (78.2%) were admitted to the hospital with advanced disease. Half of the patients had stage III disease, whereas 26.3% had stage IV disease. Ninety-five patients started their therapeutic path in our centre, whereas forty patients were transferred from other hospitals. 

Considering the type of the first intervention performed in patients, we made the following division: (1) PDS, or primary debulking surgery, (2) laparoscopy, (3) exploratory laparotomy, which resulted in a biopsy, and (4) no surgical intervention. Patients from the last group underwent further treatment, e.g., based on the results of imaging tests. Regarding the first interventions carried out in our centre, 24 patients underwent primary laparoscopy. Women who underwent PDS constituted 50.5% of the cohort; a biopsy was performed in less than one-fifth of the patients, whereas in five patients, no intervention was undertaken. When it comes to patients starting treatment in other hospitals, half of them had PDS before being transferred to the SKPP. Only 5% of women underwent primary laparoscopy; however, more than 40% of women underwent exploratory laparotomy. Only one patient was referred to SKPP without any primary surgical intervention.

Then, all patients were divided into groups depending on the final treatment decision as follows: (1) PDS—primary debulking surgery performed immediately after laparotomy and in patients for exploratory laparoscopy, (2) NACT after laparotomy—neoadjuvant treatment followed biopsy or adnexectomy in laparotomy, (3) NACT after laparoscopy—neoadjuvant treatment took place when the laparoscopic evaluation did not allow for complete cytoreductive surgery, (4) chemotherapy—concerned those patients who did not qualify for surgery, and (5) NACT without primary surgery—these patients received neoadjuvant treatment based on imaging tests without primary intervention.

Nearly 78% of women had histopathologically diagnosed high-grade serous ovarian cancer, whereas other subtypes such as LGSC, clear-cell carcinoma, endometrioid cancer, etc., were observed significantly less frequently. In the group of patients in whom we assessed the achieved cytoreduction, we considered 111 out of 135 women. Firstly, not all patients had surgical intervention in the course of treatment, and secondly, we did not manage to obtain all the operating protocols for women operated on in other hospitals. The stage of the obtained cytoreduction was most often assessed as total (46.8%), then suboptimal (29.7%), and least often, optimal (23.5%). The baseline characteristics of the groups are presented in Table 1.

During the two years of observation, more than twice as many first-time patients came to our clinic than those transferred from other centres. The groups of patients had a similar age structure and did not differ in terms of the stages of their disease. In the advanced stage, 81.1 and 83.3% of patients reported to SKPP and continued treatment, respectively. There was a significant dependency between being treated in the SKPP from the beginning of one’s diagnosis and the type of the first intervention (*p* = 0.006), and similarly, to the final decision (0.032). Among patients in the SKPP group, a more significant portion of patients underwent laparoscopy than among patients transferred from other places (25% vs. 5%), and a minor portion of patients underwent exploratory laparotomy (18.9% and 42.5%, respectively). No other dependencies were observed based on the original place of treatment, which is presented in Table 2.

When comparing patients enrolled in 2019 and 2020, according to the FIGO classification, there was a similar number of patients in both groups who had stage I/II or III/IV/non-staging of the disease. Additionally, no significant differences in the type of first intervention were found between the patients admitted in 2019 and 2020. However, regarding the final decision, the difference is already noticeable (*p* = 0.002). In 2020, more patients underwent primary cytoreductive surgery and were offered neoadjuvant treatment after laparoscopy. In contrast, fewer women had NACT after laparotomy. There were also a few women who had neoadjuvant chemotherapy without primary surgery. As far as the degree of cytoreduction is concerned, no dependencies were observed, which is shown in Table 3. 

Almost all of the univariate cox regression models for the occurrence of relapse were significant. Having the first intervention performed in a hospital other than SKPP increased the risk of relapse by 89% compared to the risk for patients treated in SKPP (*p* = 0.043). Patients with an advanced stage of OC (III, IV or non-staging FIGO stage) had an 11.84 times higher risk of relapse compared to others (*p* = 0.015). We overlooked the significant influence of the histological types of ovarian cancer on the time of the first relapse (*p* > 0.05). Compared to those from the group with PDS as the first intervention, women from the laparoscopy group had a 2.67 times higher risk of relapse (*p* = 0.017) and women from the exploratory laparotomy group had a 3.65 times higher risk of relapse (*p* < 0.001).

Additionally, having either NACT after laparotomy or after laparoscopy increased the risk of relapse 4.27 and 2.93 times, respectively (*p* < 0.001, *p* = 0.01). The stage of the cytoreduction was the last significant predictor for relapse—optimal cytoreduction compared to total cytoreduction increased the risk of relapse by 3.81 times (*p* = 0.003), and suboptimal compared to total cytoreduction increased that risk by 2.75 times (*p* = 0.023), which is presented in Table 4.

The multivariate cox regression model was built using a step method. All of the variables from the univariate models were put into the initial multivariate model. Three predictors in the multivariate cox regression model were significant. Patients from the NACT after laparotomy group compared to patients from the PDS group had a 7.10 higher risk of relapse (*p* < 0.001), and patients from the NACT after laparoscopy group compared to those from the PDS group had a 4.27 times higher risk of relapse (*p* = 0.002). Additionally, achieving optimal cytoreduction increased the risk of relapse 3.62 times in relation to total cytoreduction (*p* = 0.004). We did not detect multicollinearity between the predictors (VIF < 2.00), which is shown in Table 5.

**Figure 4:**
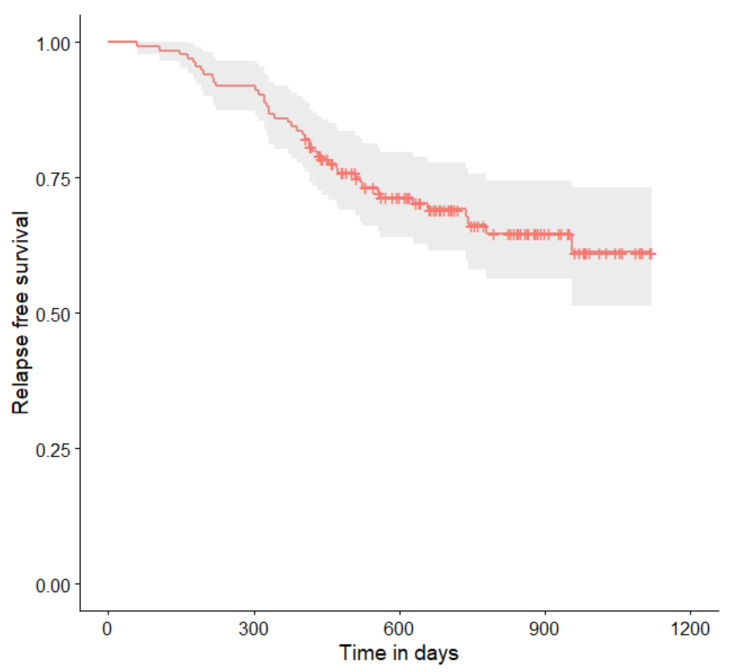
Relapse-free survival curve with 95% CI (darkened area) for all patients.

**Figure 5:**
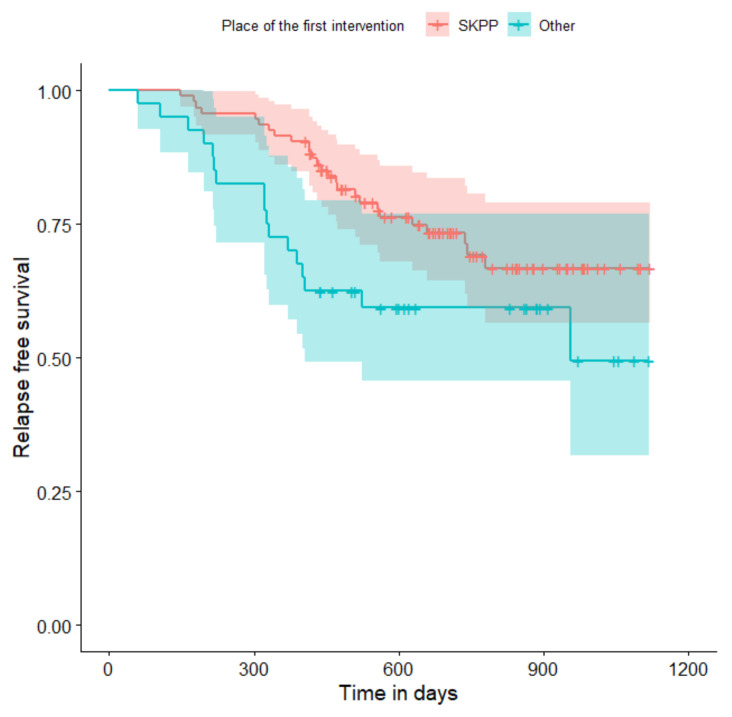
Relapse-free survival curve with 95% CI (darkened area) broken down by place of the first intervention.

**Figure 6:**
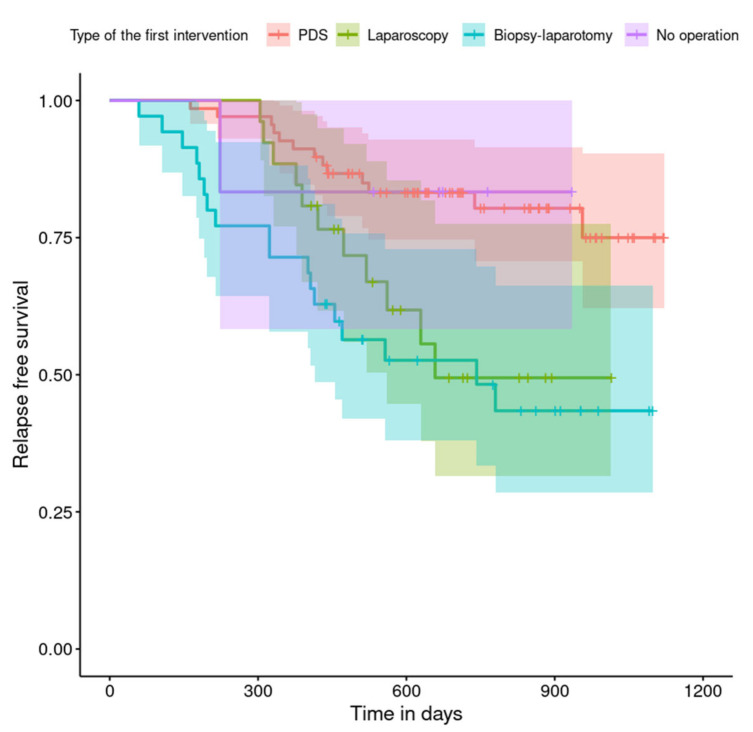
Relapse-free survival curve with 95% CI (darkened area) broken down by type of the first intervention.

**Figure 7:**
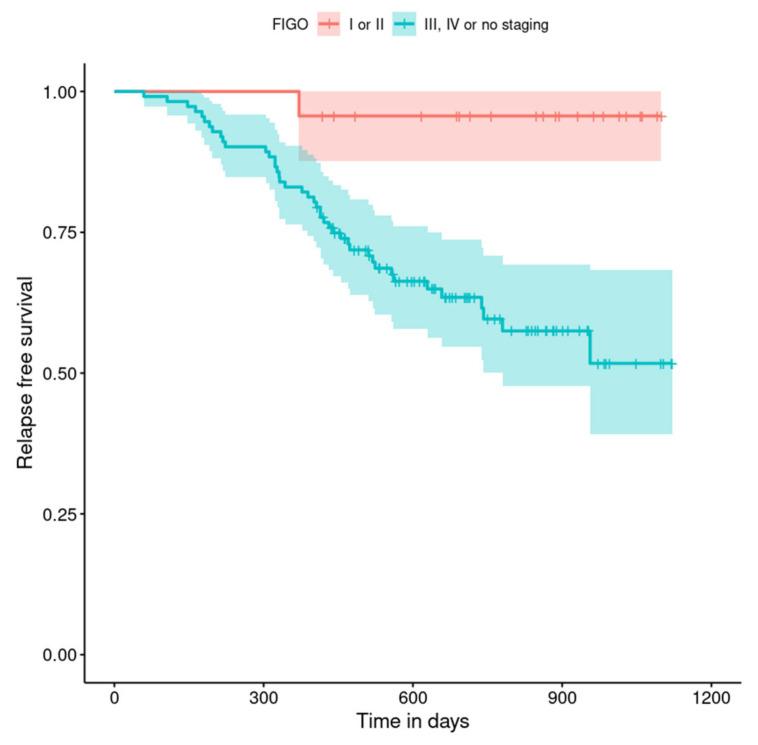
Relapse-free survival curve with 95% CI (darkened area) broken down by FIGO score.

**Figure 8:**
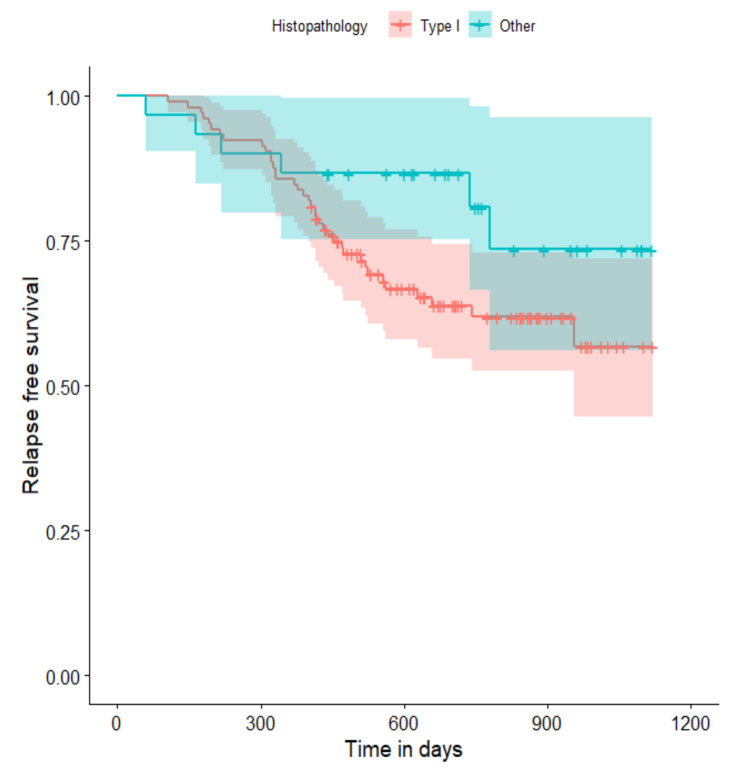
Relapse-free survival curve with 95% CI (darkened area) broken down by histopathology result (serous cancer vs. other types).

**Figure 9:**
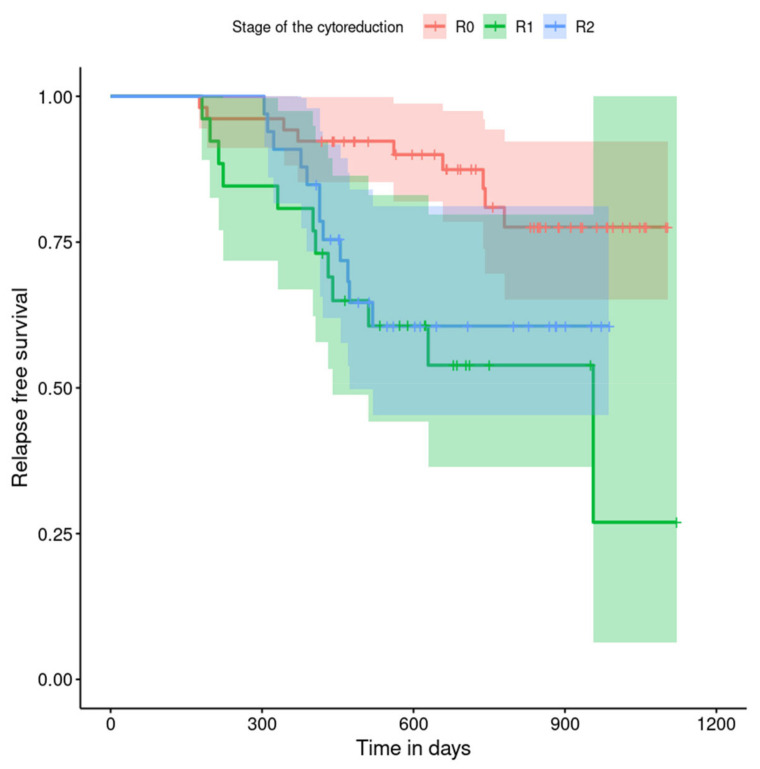
Relapse-free survival curve with 95% CI (darkened area) broken down by results of the operation.

**Figure 10:**
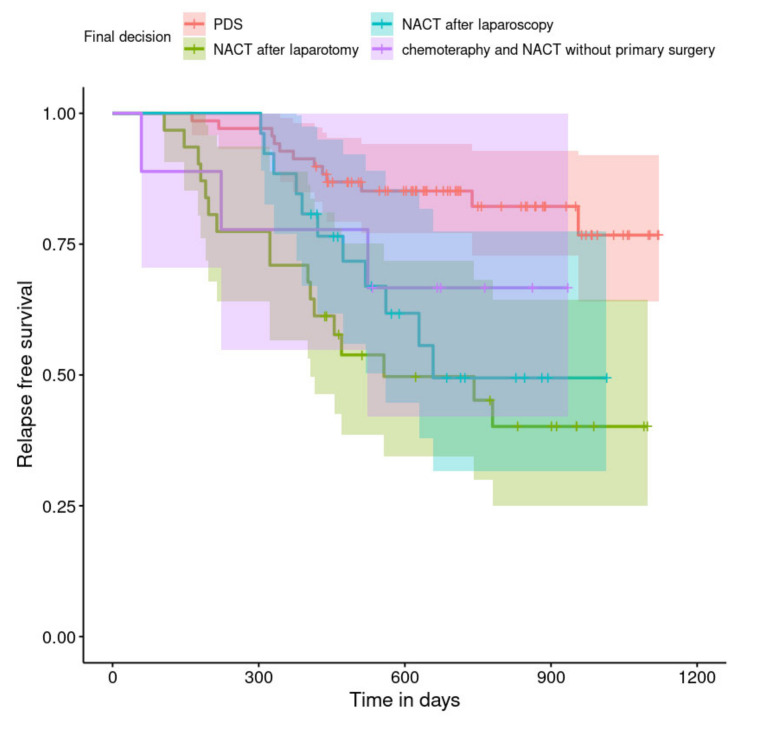
Relapse-free survival curve with 95% CI (darkened area) broken down by the final decision.

The relapse-free survival curves are shown in Figure 4, Figure 5, Figure 6, Figure 7, Figure 8, Figure 9 and Figure 10 (for all of the patients and broken down into smaller groups). The number of patients who relapsed was 43—the last patient relapsed after 956 days. The cumulative progression-free survival rate at the end of the follow-up period was 61% for all of the patients. The relapse-free survival rate for all of the patients is presented in Figure 4. The relapse rate was higher for patients treated in a place other than SKPP—the cumulative proportion of patients who did not relapse at the end of the follow-up period was 67% for SKPP patients and 50% for other patients, as shown in Figure 5. The relapse rate was higher for patients from the laparoscopy and exploratory laparotomy groups than for patients from the PDS group (*p* = 0.001). Even though patients from the group without surgery had less relapse than after neoadjuvant chemotherapy, we do not consider it to be due to the small number of people. The cumulative proportion of patients who did not relapse at the end of the follow-up period was 75% for the PDS group, 49% for the laparoscopy group, 43% for the biopsy–laparotomy group and 83% for patients that had no operation, as shown in Figure 6. Patients with stage I or II OC according to FIGO had a lower relapse rate than patients with stage III, IV or non-staging OC (*p* = 0.002); the cumulative proportion of patients who did not relapse at the end of the follow-up period was 96% for FIGO grading I or II patients and 52% for FIGO grading III or IV or non-staging patients, as shown in Figure 7. The cumulative proportion of patients who did not relapse at the end of the follow-up period was 57% for patients with serous cancer and 74% for patients with other histopathological cancer types. The relapse rate did not significantly differ between groups, as shown in Figure 8. Patients from the R1 group had the highest relapse rate, followed by patients from the R2 group, and patients from the R0 group had the lowest relapse rate (*p* = 0.005). The cumulative proportion of patients who did not relapse during the follow-up was 77% for the R0 group, 27% for the R1 group and 61% for the R2 group, as shown in Figure 9. As far as the final decision is concerned, patients from the PDS group had the lowest relapse rate, followed by patients from the chemotherapy group. Patients from the NACT after laparoscopy or laparotomy groups had a higher relapse rate (*p* < 0.001). The cumulative proportion of patients who did not relapse during the follow-up was 77% for the PDS group, 40% for the NACT after laparotomy group, 49% for the NACT after laparoscopy group and 67% for the chemotherapy and NACT without primary surgery group, as shown in Figure 10.

## 5. Discussion

The analysis presented here is from our own material regarding patients with ovarian cancer in a representative group of patients who reported to the Department of Gynaecological Oncology of the University of Medical Sciences in 2019–2020. The aim of the study was to evaluate the results of treatment and to find factors influencing the differences between patients treated from the beginning in SKPP and those transferred from other hospitals. We set progression-free survival as an end point. The analysis confirmed the superiority of treating patients from the beginning to the end of their diagnosis under one roof, and not transferring them between different centres. Having the first intervention conducted in a hospital other than SKPP increased the risk of relapse by 89%. We did not observe this relationship in the multivariate cox regression model, which may be due to slight data gaps in individual groups, which means that part of the data was truncated. In the multivariate model, only the subjects who were not missing any data for all of the entered variables were analysed, which could have influenced the significance change. We could possibly obtain a meaningful relationship in the multivariate cox regression model by testing more subjects.

Data presented by Bristow et al. were used in the most extensive comparative analysis of ovarian cancer treatment across centres. The researchers included 7272 patients in their project, which significantly increased the reliability of the trial. However, factors such as race, socioeconomic status (SES) and the type of patient’s insurance were analysed, which we did not consider due to the homogeneous group of patients and the lack of differences between patients in this respect in our study. In our study, all of the patients were treated using public funds. However, it seems that some patients who live further away from the oncological unit may experience longer waiting times for admission and surgery due to individual logistical difficulties. In addition, these patients may be guided by the hospital’s proximity and not by the hospital’s volume and the number of procedures performed there.

The median ovarian-cancer-specific survival rate for all of the patients in the study provided by Bristow was 28.2 months. This suggests that we should extend the observation time in relation to the survival time of the patients.

The body of health services research regarding volume–outcome relationships for cancer care convincingly indicates that the benefit from being cared for at high-volume centres exceeds the benefit from breakthrough treatments and merits efforts to concentrate initial care for all forms of cancer [3]. Disparities in access to high-volume health care providers and hospitals have been described according to race, ethnicity and sociodemographic characteristics for different types of cancer, such as breast, colorectal, gastric and lung cancer, as well as cardiovascular disease and orthopaedic conditions [4,5]. Regarding ovarian cancer, a consistent volume–outcome relationship has been well documented by multiple population-based and single-institution studies, which showed that superior treatment and survival outcomes are associated with surgical expertise and multidisciplinary resources offered by high-volume surgeons and high-volume hospitals [6,7,8,9,10,11,12,13,14,15]. Disparities in ovarian cancer survival rates associated with race and SES are, therefore, thought to be largely due to unequal access to care and the administration of non-standard treatment regimens, although genetic susceptibility and a higher frequency of modifiable risk factors cannot be excluded as causative factors. In a review of the global literature, Chornokur et al. concluded that unequal access to care is primarily a consequence of lower SES and a lack of private health insurance among minority populations [16]. Indeed, single-institution and cooperative group trial studies have shown that when access to speciality providers at high-volume centres is provided equally, and all patients receive comparable treatment, racial disparities in ovarian cancer survival rates are largely mitigated.

As expected, the percentages of histological types of ovarian cancer show that the most common is high-grade serous ovarian cancer, reflecting the distribution of histological types worldwide. These data align with global reports that most ovarian epithelial carcinomas are serous. High-grade serous ovarian cancers are characterised by a high level of malignancy and are usually recognised as significantly advanced [17,18]. Similarly, according to the FIGO classification, the percentage of patients presenting with progressive disease at stages III, IV and non-staging shows an accurate clinical picture of patients with ovarian cancer. Most first-time patients are diagnosed with advanced and disseminated diseases.

Our results reveal that in 2020, compared to in 2019, noticeably more operations starting with laparoscopy were performed as the first intervention. In the presented group, based on the operating protocols and available medical documentation, significantly more patients who started treatment in SKPP underwent laparoscopy before laparotomy. This indicates that more patients qualified for neoadjuvant treatment. Interestingly, patients transferred from other hospitals were admitted significantly more often after burdensome explorative laparotomies. Not only is the type of first intervention important, but also the overall rate resulting in the final decision on the proposed treatment. The data we have gathered show that patients undergoing neoadjuvant chemotherapy have a higher risk of relapse than those primarily, successfully operated on. To be exact, patients treated with NACT after exploratory laparotomy had over a four times higher relapse rate and those treated with NACT after laparoscopy had almost a three times higher relapse rate than the PDS group. This is not in line with the results of the report by Vergote et al. that found that neoadjuvant chemotherapy followed by interval debulking surgery (IDS) was not inferior to PDS followed by chemotherapy as a treatment option for patients with advanced OC (stage IIIC or IV). The complete resection of all macroscopic lesions, whether performed as PDS or after NACT, remains the objective whenever cytoreductive surgery is performed [19].

There is no doubt that the best treatment results, and thus, longer relapse-free time, are achieved by the originally performed debulking surgery. However, this is the case only if the result of this operation is total cytoreduction. What is more, the influence of the scope of resection on OS is unquestionable. In 2009, it was proved by du Bois [20] that after radical surgery, in which total resection was achieved, i.e., R0, 50% of patients’ overall survival rate was over 90 months, in contrast to optimal (R1) and suboptimal (R2) resection, where the survival rate of 50% of patients was similar and was slightly more than 36 months. Our pilot study was too short to assess the impact of resectability, neither on 5-year nor overall survival rates. However, this might be a topic for further observation, and we should extend the research time. Interestingly, our study showed that patients from the R1 group had the highest relapse rate. The cumulative proportion of patients who did not relapse at the end of the follow-up period was 27% for the R1 group and 66% for R2. An incorrect assessment of optimal resection might explain the observed relationship. This may be because we analysed patients operated upon in various medical centres characterised by a different experience of oncological surgery, and perhaps not all surgeons rated the obtained resectability in the same way.

Patients who cannot obtain at least optimal cytoreduction during either qualification for surgery or the resection assessment are candidates for neoadjuvant chemotherapy. It is worth noting that a suboptimal procedure significantly reduces the time to progression and the overall survival rate. Therefore, the inability to achieve total/optimal cytoreduction should be studied in detail [21].

Another conclusion emerging from our analysis is that patients benefit the most from surgery in a centre with more experience in treating ovarian cancer. Bristow et al. confirmed that among patients with advanced ovarian cancer, the provision of a combination of high-volume hospitals and high-volume physicians (HVPs) is an independent predictor of an improved disease-specific survival rate [22]. Although it was conducted using a smaller cohort of patients, our study drew similar conclusions. We believe that survival rate is influenced not only by the operator’s technical skills, but also by a holistic, systemic approach to patients. The body of health services research regarding volume–outcome relationships for cancer care convincingly indicates that the great benefit from care at a HVH exceeds the one from breakthrough treatments and merits efforts to concentrate care for ovarian cancer.

## 6. Conclusions

The above work compares the results of the treatment of patients in one centre and those migrating between hospitals. The obtained conclusions show that it is worth referring patients to high-volume hospitals because it is possible to make the best therapeutic decision for patients in primarily inoperable situations. Based on laparoscopy, patients may be referred either to PDS or neoadjuvant chemotherapy. Soon, laparoscopy may become the standard decision made for suspected advanced cancers. As a less invasive procedure, it reduces the time to administer NACT chemotherapy. PDS that is initially performed and achieves total cytoreduction remains the best in treating ovarian cancer, as underlined by world data and this work. However, it should be borne in mind that not every patient is treated in the best centres, but we, as doctors, should manage their treatment in the best possible way.

### 6.1. Limitations of the Study

The major limitation of this study is the relatively small number of subjects who participated in the observation and the relatively short time frame. The main reasons for this were the rigorous inclusion and exclusion criteria. Conversely, such criteria enabled us to select a homogenous group of subjects. However, we will be able to expand the group by using data from patients treated before 2019 and analyse larger cohorts of patients in the future. This might enable us to update the rates of overall survival (OS), objective response rate (ORR) and progression-free survival (PFS).

### 6.2. Strengths of the Study

It is worth noting that in the analysed research, we collected a homogeneous and representative research group and compared various parameters. The inclusion and exclusion criteria were strict, which eliminated the influence of confounders. Additionally, the large share of subjects transferred to our centre is an advantage. The study’s greatest strength is its comparative nature, which allowed for a conclusion to be drawn about the benefits of holistic treatment in one centre. This approach has only been used in a few previous studies.

## Figures and Tables

**Table 1:** Baseline characteristics of the study group.

Characteristic	Value, *n* (%)
Year of measurement	
2019	67 (49.6)
2020	68 (50.4)
Place of the first intervention	
SKPP	95 (70.4)
other hospitals	40 (29.6)
Type of the first intervention	
SKPP	
PDS–laparotomy	48 (50.5)
laparoscopy	24 (25.3)
Biopsy–laparotomy	18 (18.9)
no operation	5 (5.3)
Other hospitals	
PDS–laparotomy	20 (50.0)
laparoscopy	2 (5.0)
Biopsy–laparotomy	17 (42.5)
no operation	1 (2.5)
Final decision	
PDS after laparotomy or laparoscopy	69 (51.0)
NACT after laparotomy	31 (23.0)
NACT after laparoscopy	26 (19.3)
chemotherapy	5 (3.7)
NACT without primary surgery	4 (3.0)
FIGO staging system	
I	17 (12.6)
II	6 (4.5)
III	69 (51.1)
IV	35 (25.9)
non-staging	8 (5.9)
Histopathology	
HGSC	105 (77.8)
other types	30 (22.2)
Stage of the cytoreduction (*n* = 111)	
total (R0)	52 (46.8)
optimal (R1)	26 (23.5)
suboptimal (R2)	33 (29.7)

PDS—primary debulking surgery; NACT—neoadjuvant chemotherapy; HGSC—high-grade serous carcinoma.

**Table 2:** Comparison of selected characteristics between patients from SKPP and those transferred from other centres.

Characteristic, *n* (%)	SKPP Patients	Transferred Patients	*p*
FIGO			
I or II	17 (17.9)	6 (15.0)	0.828
III or IV	73 (76.8)	31 (77.5)	
non-staging	5 (5.3)	3 (7.5)	
Type of the first intervention			
PDS–laparotomy	48 (50.5)	20 (50.0)	0.006
laparoscopy	24 (25.3)	2 (5.0)	
Biopsy–laparotomy	18 (18.9)	17 (42.5)	
no operation	5 (5.3)	1 (2.5)	
Final decision			
PDS after laparoscopy and laparotomy	48 (50.5)	21 (52.5)	
NACT after laparotomy	18 (18.9)	13 (32.5)	
NACT after laparoscopy	24 (25.3)	2 (5.0)	0.032
chemotherapy	2 (2.1)	3 (7.5)	
NACT without primary surgery	3 (3.2)	1 (2.5)	
Stage of the cytoreduction			
total (R0)	41 (46.6)	11 (47.8)	0.539
optimal (R1)	19 (21.6)	7 (30.4)	
suboptimal (R2)	28 (31.8)	5 (21.7)	

Dependencies between the group and selected characteristics were analysed using the chi-square test with Yates’ correction for continuity for two categorical variables. FIGO—the International Federation of Gynaecology and Obstetrics; PDS—primary debulking surgery; NACT—neoadjuvant chemotherapy; *p*—*p*-value.

**Table 3:** Comparison of selected characteristics between subjects admitted in the years 2019 and 2020.

Characteristic, *n* (%)	2019 Group	2020 Group	*p*
FIGO staging system			
I or II	14 (20.9)	9 (13.2)	0.420
III or IV	50 (74.6)	54 (79.4)	
Non-staging	3 (4.5)	5 (7.4)	
Type of the first intervention			
PDS–laparotomy	31 (46.3)	37 (54.4)	0.060
laparoscopy	10 (14.9)	16 (23.5)	
Biopsy–laparotomy	24 (35.8)	11 (16.2)	
no operation	2 (3.0)	4 (5.9)	
Final decision			
PDS after laparoscopy and laparotomy	30 (44.8)	39 (57.4)	
NACT after laparotomy	22 (32.8)	9 (13.2)	
NACT after laparoscopy	10 (14.9)	16 (23.5)	0.002
chemotherapy	5 (7.5)	0 (0.0)	
NACT without primary surgery	0 (0.0)	4 (5.9)	
Place of the first intervention			
SKPP	43 (64.2)	52 (76.5)	0.169
Other hospitals	24 (35.8)	16 (23.5)	
Stage of the cytoreduction			
total (R0)	27 (54.0)	25 (41.0)	0.208
optimal (R1)	8 (16.0)	18 (29.5)	
suboptimal (R2)	15 (30.0)	18 (29.5)	

FIGO—the International Federation of Gynaecology and Obstetrics; PDS—primary debulking surgery; NACT—neoadjuvant chemotherapy; *p*—*p*-value. Dependencies between the group and selected characteristics were analysed using the chi-square test with Yates’ correction for continuity for two categorical variables.

**Table 4:** The influence of individual factors on the occurrence of relapse using the univariate cox regression model.

Variable	HR	95% CI	*p*
Place of the first intervention (SKPP vs. other hospitals)	1.89	1.02; 3.48	0.043
FIGO (I or II vs. III, IV or non-staging)	11.84	1.62; 86.32	0.015
Histopathology (HGSC vs. other)	0.50	0.21; 1.18	0.111
Type of the first intervention (PDS vs. …)			
laparoscopy	2.67	1.19; 5.99	0.017
Biopsy–laparotomy	3.65	1.79; 7.47	<0.001
no operation	0.95	0.12; 7.26	0.959
Type of the first intervention (laparoscopy vs. …)			
Biopsy–laparotomy	1.37	0.64; 2.90	0.415
no operation	0.35	0.05; 2.75	0.321
Final decision (PDS vs. …)			
NACT after laparotomy	4.27	2.04; 8.97	<0.001
NACT after laparoscopy	2.93	1.29; 6.67	0.010
chemotherapy or NACT without primary surgery	2.26	0.64; 8.04	0.207
Stage of the cytoreduction (total—R0 vs. …)			
optimal (R1)	3.81	1.59; 9.12	0.003
suboptimal (R2)	2.75	1.15; 6.57	0.023

FIGO—the International Federation of Gynaecology and Obstetrics; HGSC—high-grade serous carcinoma; PDS—primary debulking surgery; NACT—neoadjuvant chemotherapy; LAP—laparoscopy; *p*—*p*-value; HR—hazard ratio; HR with a 95% CI—hazard ratio with a 95% confidence interval for the occurrence of relapse.

**Table 5:** The influence of all variables on the occurrence of relapse using the multivariate cox regression model.

Variable	HR	95% CI	*p*
Final decision (PDS vs. …)			
NACT after laparotomy	7.10	2.82; 17.92	<0.001
NACT after laparoscopy	4.27	1.68; 10.85	0.002
Chemotherapy or NACT without primary surgery	1.91	0.24; 15.41	0.546
Stage of the cytoreduction (R0 vs. …)			
R1	3.62	1.48; 8.84	0.004
R2	1.70	0.69; 4.17	0.248

HR with a 95% CI—hazard ratio with a 95% confidence interval for the occurrence of relapse; FIGO—the International Federation of Gynaecology and Obstetrics; PDS—primary debulking surgery; NACT—neoadjuvant chemotherapy; *p*—*p*-value; HR—hazard ratio.

## Data Availability

All data are available from the corresponding author.
